# Genomic prediction of piglet response to infection with one of two porcine reproductive and respiratory syndrome virus isolates

**DOI:** 10.1186/s12711-018-0371-4

**Published:** 2018-02-01

**Authors:** Emily H. Waide, Christopher K. Tuggle, Nick V. L. Serão, Martine Schroyen, Andrew Hess, Raymond R. R. Rowland, Joan K. Lunney, Graham Plastow, Jack C. M. Dekkers

**Affiliations:** 1 Department of Animal Science, Iowa State University, Ames, IA USA; 2 College of Veterinary Medicine, Kansas State University, Manhattan, KS USA; 3 USDA, ARS, BARC, Beltsville, MD USA; 4 University of Alberta, Edmonton, AB Canada

## Abstract

**Background:**

Genomic prediction of the pig’s response to the porcine reproductive and respiratory syndrome (PRRS) virus (PRRSV) would be a useful tool in the swine industry. This study investigated the accuracy of genomic prediction based on porcine SNP60 Beadchip data using training and validation datasets from populations with different genetic backgrounds that were challenged with different PRRSV isolates.

**Results:**

Genomic prediction accuracy averaged 0.34 for viral load (VL) and 0.23 for weight gain (WG) following experimental PRRSV challenge, which demonstrates that genomic selection could be used to improve response to PRRSV infection. Training on WG data during infection with a less virulent PRRSV, KS06, resulted in poor accuracy of prediction for WG during infection with a more virulent PRRSV, NVSL. Inclusion of single nucleotide polymorphisms (SNPs) that are in linkage disequilibrium with a major quantitative trait locus (QTL) on chromosome 4 was vital for accurate prediction of VL. Overall, SNPs that were significantly associated with either trait in single SNP genome-wide association analysis were unable to predict the phenotypes with an accuracy as high as that obtained by using all genotyped SNPs across the genome. Inclusion of data from close relatives into the training population increased whole genome prediction accuracy by 33% for VL and by 37% for WG but did not affect the accuracy of prediction when using only SNPs in the major QTL region.

**Conclusions:**

Results show that genomic prediction of response to PRRSV infection is moderately accurate and, when using all SNPs on the porcine SNP60 Beadchip, is not very sensitive to differences in virulence of the PRRSV in training and validation populations. Including close relatives in the training population increased prediction accuracy when using the whole genome or SNPs other than those near a major QTL.

## Background

Improving phenotypic performance of livestock is the overall goal of animal breeding programs. For some economically important traits, phenotypes on selection candidates or close relatives are difficult to obtain due to high cost of phenotyping, strict biosecurity measures, or age at which phenotypes are measurable. Genomic prediction provides an attractive alternative to select for these traits. Genomic prediction involves the use of genotypes at single nucleotide polymorphisms (SNPs) across the genome to predict phenotypes that have not been observed in the selection candidates [1]. SNP chips that contain thousands to hundreds of thousands of genetic markers that cover the whole genome are now available for most livestock species [2].

Porcine reproductive and respiratory syndrome (PRRS) is an economically devastating disease in the swine industry caused by a rapidly mutating virus (PRRSV) [3]. Genomic prediction for response to the PRRS virus (PRRSV) in pigs would be highly valuable to the swine industry, as most selection takes place in high health nucleus farms that are unlikely to face PRRSV outbreaks. Conducting experimental PRRSV infection trials requires the use of strictly regulated biocontainment facilities and is expensive and labor intensive. Therefore, the ability to combine data from pigs with different genetic backgrounds that were infected with different PRRSV isolates to use as a training population for genomic prediction of response to other PRRSV isolates of unrelated piglets would be very beneficial.

The data used in this study were from the PRRS Host Genetics Consortium (PHGC) [4] and included phenotypes on weight gain (WG) and viral load (VL) from nine trials of ~ 200 piglets that were infected with the NVSL 97-7985 (NVSL) PRRSV isolate [5] and from four similarly sized trials of piglets infected with the KS2006-72109 (KS06) isolate. Using data from the first eight NVSL trials, Boddicker et al. [6–8] discovered and validated a quantitative trait locus (QTL) on *Sus scrofa* chromosome (SSC) 4 for VL and WG and showed that a single SNP in this region, WUR10000125 (WUR), explained most of the genetic variance of the QTL. Furthermore, using the NVSL data, Boddicker et al. [8] showed that the accuracy of genomic prediction across breeds was maximized when only the SNPs within the 1-Mb window containing the WUR SNP were used. Our investigation expands these questions to prediction across PRRSV isolates. Hess et al. [9] showed that genotype at the WUR SNP was associated with VL for both PRRSV isolates but with WG only for the NVSL isolate. Genetic correlations for VL and WG between the two isolates were both estimated to be 0.86, which indicates that accurate genomic prediction across isolates should be possible. Also, using field data, which likely contained multiple PRRSV isolates or strains of infection, Serão et al. [10] showed that genomic prediction of PRRSV antibody response was moderately accurate. These findings are important to the swine industry, as PRRSV is a rapidly mutating virus [3, 11] and different strains are infecting industry populations and are likely to evolve from one outbreak to the next.

Except for the SSC4 QTL, which was associated with VL in both isolates, genome-wide association studies (GWAS) in the data used in this study did not identify genomic regions that overlapped between the two PRRSV isolate datasets [12], in spite of the high genetic correlations that have been estimated for traits between isolates [13]. However, although the most strongly associated regions were inconsistent between these two PRRSV isolates, we found that genes near these SNPs were enriched for several of the same gene ontology (GO) terms.

Against this background, the objectives of this study were to assess the accuracy of genomic prediction for VL and WG in the following scenarios: (1) across PRRSV isolates and genetic sources and (2) using all SNPs, all SNPs other than those in the SSC4 QTL region, or only the WUR SNP.

## Methods

All experimental protocols used in this study were approved by the Kansas State University (KSU) Animal Care and Use Committee.

### Study design and animal populations

Lunney et al. [4] provided a detailed description of the study design employed in the PHGC trials used in this study. Briefly, 13 trials of approximately 200 commercial crossbred piglets each were sent to Kansas State University at weaning, given 1 week to acclimate, then inoculated intramuscularly and intranasally with 10^5^ TCID50 of either the NVSL 97-7985 (NVSL) [5] or KS2006-72109 (KS06) PRRSV isolate. Blood samples were collected at 0, 4, 7, 11, and 14 days post-infection (dpi) and then weekly until termination of the trial at 42 dpi. Individual weights were observed weekly throughout the trial. At 42 dpi, piglets were euthanized and ear tissue was collected for genomic DNA extraction, which was sent to GeneSeek, Inc. (trials 1–10; Lincoln, Nebraska, USA) or Delta Genomics (trials 11–15; Edmonton, Alberta, CA) and genotyped using the Illumina Porcine SNP60 Beadchip [2]. Quality control of genotype data has been previously described [12]. Briefly, after filtering genotypes with GenCall scores lower than 0.5, minor allele frequencies less than 0.01, and genotyping call rates less than 0.80, 52,386 SNP remained, with an overall genotyping rate of 99.2%.

**Fig. 1:**
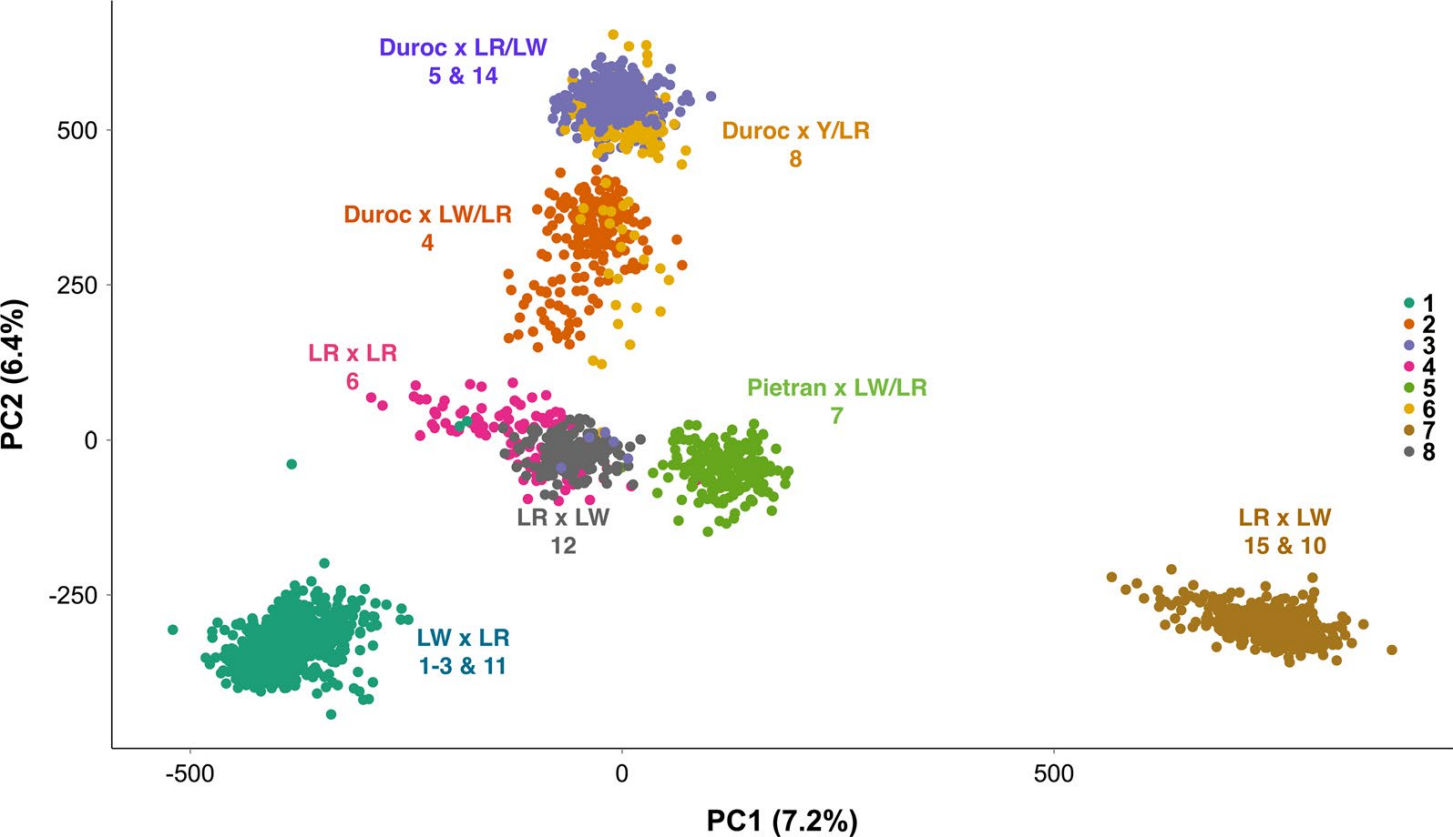
Principal components analysis of the SNP genotype data. Each point represents a single animal, with each color representing one of the eight genetic lines used in this study. The numbers for each genetic line match those shown in Table 1. The breed makeup of the animals for each genetic line is shown in the same color. *LR* landrace, *LW* large white, and *Y* yorkshire. Breeds are presented as breed of sire × breed of dam

In total, data on 2288 commercial crossbred piglets from eight genetic backgrounds were used. Piglets averaged 26.6 (± 2.4) days of age and weighed 7.17 (± 1.4) kg at the time of infection. Of this total, 1557 piglets were infected with NVSL, with an average weight of 7.34 (± 1.4) kg and 26.6 (± 2.6) days of age at inoculation. The remaining 731 piglets averaged 26.7 (± 1.9) days of age and weighed 6.80 (± 1.29) kg on average at the time of inoculation with KS06. A more detailed description of the populations used in each trial is in Waide et al. [12]. Trial 9 involved pigs from the ISU RFI selection lines [14] and were excluded from these analyses. Trial 13 was also excluded from these analyses because piglets from this trial had much lower and more variable viremia profiles compared to the other KS06 trials. Relationships between piglets used in these trials were investigated using principal component analysis (PCA) of genotypes for all SNPs using the R function prcomp [15], as shown in Fig. 1.

### Phenotypes

The two phenotypes analyzed in this study were described by Boddicker et al. [6]. Briefly, the amount of PRRSV RNA in blood samples was estimated using quantitative PCR and reported as the log_10_ of PRRSV RNA copies relative to a standard curve. Viral load (VL) was calculated as the area under the curve of viremia up to and including 21 dpi. WG was calculated as the difference between body weight at 42 and 0 dpi.

### Genomic prediction analyses

Bayesian variable selection, Bayes-B, was used to estimate additive and dominance effects for 52,268 SNPs for genomic prediction using the following model:

$${\mathbf{y}} = {\mathbf{Xb}} + \mathop \sum \limits_{i = 1}^{k} {\mathbf{z}}_{{\varvec{ai}}} a_{i} \delta_{ai} + \mathop \sum \limits_{i = 1}^{k} {\mathbf{z}}_{{\varvec{di}}} d_{i} \delta_{di} + {\mathbf{e}},$$

where ${\mathbf{y}}$ = vector of phenotypic observations, ${\mathbf{X}}$ = incidence matrix relating phenotypes to fixed effects, ${\mathbf{b}}$ = vector of fixed effects of sex, the interactions of pen and parity with trial, genotype at the WUR SNP, and covariates of initial age and weight, ${\mathbf{z}}_{{\varvec{ai}}}$ = vector of the additive genotype covariates coded as − 10, 0, and 10 for the *AA*, *AB*, and *BB* genotypes, respectively, for SNP $i$, $a_{i}$ = additive effect for SNP $i$, $\delta_{ai}$ = indicator for whether the additive effect of SNP *i* was included ($\delta_{ai}$ = 1) or excluded from ($\delta_{ai}$ = 0) the model for a given iteration of the Markov chain Monte Carlo (MCMC) chain, ${\mathbf{z}}_{{\varvec{di}}}$ = vector of the dominance genotype covariates coded as 10, 0, and 10 for the *AA*, *AB*, and *BB* genotypes, respectively, for SNP $i$, $d_{i}$ = dominance effect for SNP $i$, $\delta_{di}$ = indicator for whether the dominance effect of SNP $i$ was included ($\delta_{di} = 1$) or excluded from ($\delta_{di} = 0$) the model for a given iteration of the MCMC chain, and ${\mathbf{e}}$ = vector of residual errors. The prior probability that a given SNP was excluded from the model ($\delta_{ai} = 0$ and $\delta_{di} = 0$) was set equal to π = 0.99, fitting approximately 1% or 523 additive effects and 523 dominance effects in each of 51,000 iterations of the MCMC chain, with the first 1000 iterations designated as burn in. In all analyses, genotype at the WUR SNP was included as a fixed effect and SNPs within 2.5 Mb on either side of the WUR SNP were excluded from the model.

Using the estimates obtained from the Bayes-B analysis, the genomic estimated genotypic value (GEGV) for the whole genome, excluding the WUR region (Genome-WUR), was obtained for each animal in the validation population using the following equation:

$$GEGV_{j} = \mathop \sum \limits_{i = 1}^{k} {\mathbf{z}}_{{\varvec{aij}}} \hat{a}_{i} + \mathop \sum \limits_{i = 1}^{k} {\mathbf{z}}_{{\varvec{dij}}} \hat{d}_{i} ,$$

where $GEGV_{j}$ = the genotypic value for individual $j$, ${\mathbf{z}}_{{\varvec{aij}}}$ and ${\mathbf{z}}_{{\varvec{dij}}}$ are as described above, $\hat{a}_{i}$ = estimate of the additive effect for SNP *i*, and $\hat{d}$_*i*_ = estimate of the dominance effect for SNP $i$. To estimate the prediction accuracy of GEGV based on genotype at the SSC4 QTL alone, we used estimates of the fixed effect for the WUR SNP from the Bayes-B analysis of the training population to estimate GEGV in the validation population. To estimate the predictive accuracy of the whole genome, we used the sum of the Genome-WUR GEGV and the GEGV based on the fixed effect estimates for the WUR SNP.

### Training and validation datasets

**Fig. 2:**
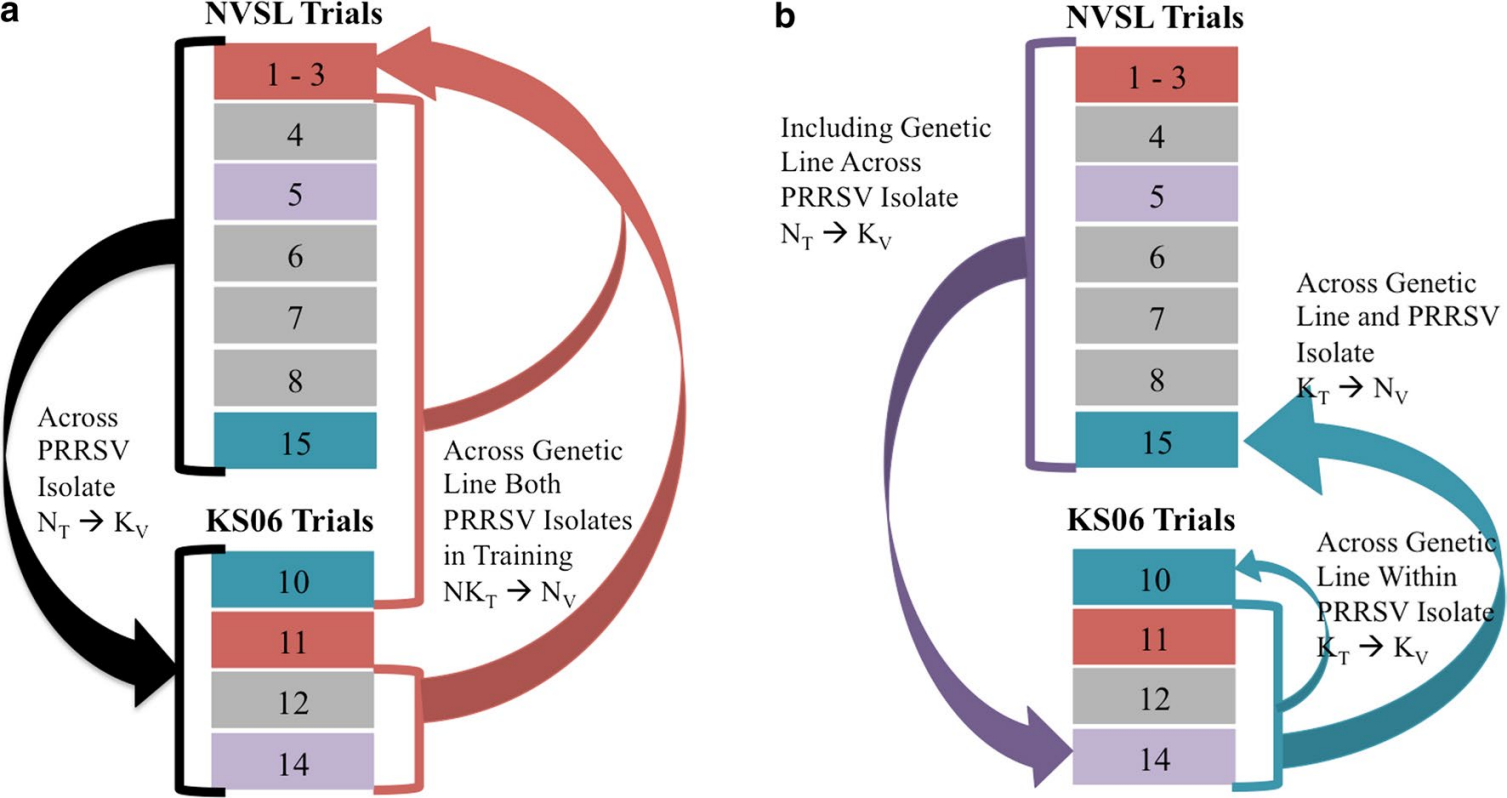
Scenarios used for training and validation for genomic prediction. Pink, purple, and blue colored rectangles represent each of the three genetic lines with pigs in both NVSL and KS06 trials, with the trial number indicated inside the rectangle. Gray colored rectangles represent individual genetic lines with only one trial of PRRSV infected pigs. Arrows indicate direction of genomic prediction, with the tail originating from the trials used in training and the head pointing towards the trial(s) used for validation. **a** Genomic prediction across PRRSV isolates using all data indicated by black arrows; across breeding company using both PRRSV isolates in training indicated by red arrows. **b** Genomic prediction across PRRSV isolate including validation breeding company in training indicated by purple arrow; genomic prediction across genetic line within PRRSV isolate and across genetic lines and isolate indicated by small and large blue arrows, respectively

Figure 2 shows examples of how the data were split into training and validation groups for the five main genomic prediction scenarios described in the following, followed by an example of each scenario: (1) genomic prediction across PRRSV isolate (black arrows in Fig. 2a): e.g., all data from the NVSL isolate used for training and all data from the KS06 isolate used for validation (N_T_ → K_V_); (2) genomic prediction across genetic line with both PRRSV isolates in training (red arrow in Fig. 2a): e.g., trials 4–8, 15 from the NVSL isolate and trials 10, 12, and 14 from the KS06 isolate used for training and trials 1–3 from the NVSL isolate used for validation (NK_T_ → N_V_); (3) genomic prediction across genetic line within PRRSV isolate (small blue arrow in Fig. 2b): e.g., trials 11, 12, and 14 from the KS06 isolate used for training and trial 10 from the KS06 isolate used for validation (K_T_ → K_V_);. (4) genomic prediction across genetic line and PRRSV isolate (large blue arrow in Fig. 2b): e.g., trials 11, 12, and 14 from the KS06 isolate used for training and trial 15 from the NVSL isolate used for validation (K_T_ → N_V_); (5) including genetic line across PRRSV isolate (purple arrow in Fig. 2b): e.g., trials 1–8 and 15 from the NVSL isolate used for training and trial 14 from the KS06 isolate used for validation (N_T_ → K_V_).

In order to determine the contribution of genetic relationships between animals in the training and validation datasets, the accuracy of genomic prediction across PRRSV isolates when validating on data from one genetic line was compared by including or excluding the genetic line used for validation from the training data (purple and large blue arrow in Fig. 2b, respectively). These analyses were only conducted for genetic lines that had data for both PRRSV isolates (five trials for the NVSL data and three trials for the KS06 data; Fig. 2).

Accuracy of genomic prediction was calculated as the correlation between the GEGV and phenotypes adjusted for the fixed effects included in the genomic prediction model, divided by the square root of the heritability of the trait for the PRRSV isolate of infection in the validation population, as estimated by Hess et al. [9], which were 0.31 and 0.40 for VL and 0.30 and 0.24 for WG in NVSL and KS06, respectively. For example, when we trained on VL from one or more NVSL infection trials and validated on VL from one or more KS06 infection trials, genomic prediction accuracy was calculated as the correlation between the GEGV and adjusted phenotypes for VL in the KS06 trial(s) divided by the square root of the estimate of heritability for VL obtained by Hess et al. [13] using all KS06 data. Phenotypes were adjusted for estimates of fixed effects (sex, interactions of pen and parity with trial, PRRSV isolate, and covariates beginning age and weight; genotype at the WUR SNP was included as a fixed effect for the Genome-WUR analyses only) within the validation population using a fixed effects only model in R (R function lm) [15].

## Results

The data used in this study consisted of 2288 pigs from eight genetic lines that were infected with one of two PRRSV isolates. Information on each trial is in Table 1. Principal component analysis (PCA) of SNP genotypes from all animals used in this study clustered pigs from each genetic line together (Fig. 1). The first principal component (PC) explained 7.2% of the variance in genotypes and PC2 explained 6.4%. PC1 distinguished pigs from genetic line 1 from pigs from genetic line 7, and each of these lines from pigs in the other genetic lines. PC2 separated pigs based on their breed composition, separating progeny of Duroc sires from those of other sires. This breed separation agrees with previous reports that show clustering of Large White (LW), Landrace (LR), and Pietrain pigs together and separate from Duroc pigs based on genomic data [16].

**Table 1:** General information on each PRRSV infection trial

Trial(s)	Genetic line	Breed	Viral load		Weight gain		PRRSV isolate
			*N*	Average (s.d.)	*N*	Average (s.d.)	
1–3	1	LW^a^ × LR^b^	504	108.3 (8.1)	487	12.1 (4.4)	NVSL
4	2	Duroc × LW/LR	192	113.2 (6.3)	190	15.9 (4.0)	
5	3	Duroc × LR/LW	184	101.4 (7.2)	183	19.1 (2.9)	
6	4	LR × LR	123	109.6 (8.0)	106	14.8 (5.6)	
7	5	Pietrain × LW/LR	189	104.5 (6.2)	189	14.5 (3.2)	
8	6	Duroc × Y^c^/LR	188	107.9 (6.6)	182	10.2 (4.6)	
15	7	LR × LW	171	107.6 (10.9)	165	19.1 (4.0)	
NVSL	Total	–	1551	107.0 (8.4)	1502	14.9 (5.0)	
10	7	LR × LW	174	93.9 (6.7)	179	19.1 (4.2)	KS06
11	1	LW × LR	170	100.4 (6.4)	178	18.6 (4.4)	
12	8	LR × LW	171	104.7 (6.3)	170	19.0 (4.1)	
14	3	Duroc × LR/LW	180	98.6 (7.7)	171	21.3 (4.1)	
KS06	Total	–	695	99.4 (7.8)	641	19.5 (4.3)	
Total	–	–	2246	104.6 (8.9)	2200	16.4 (5.2)	–

^a^Large white (LW)

^b^Landrace (LR)

^c^Yorkshire (Y)

### Genomic prediction of viral load (VL)

#### Prediction across PRRSV isolates

**Fig. 3:**
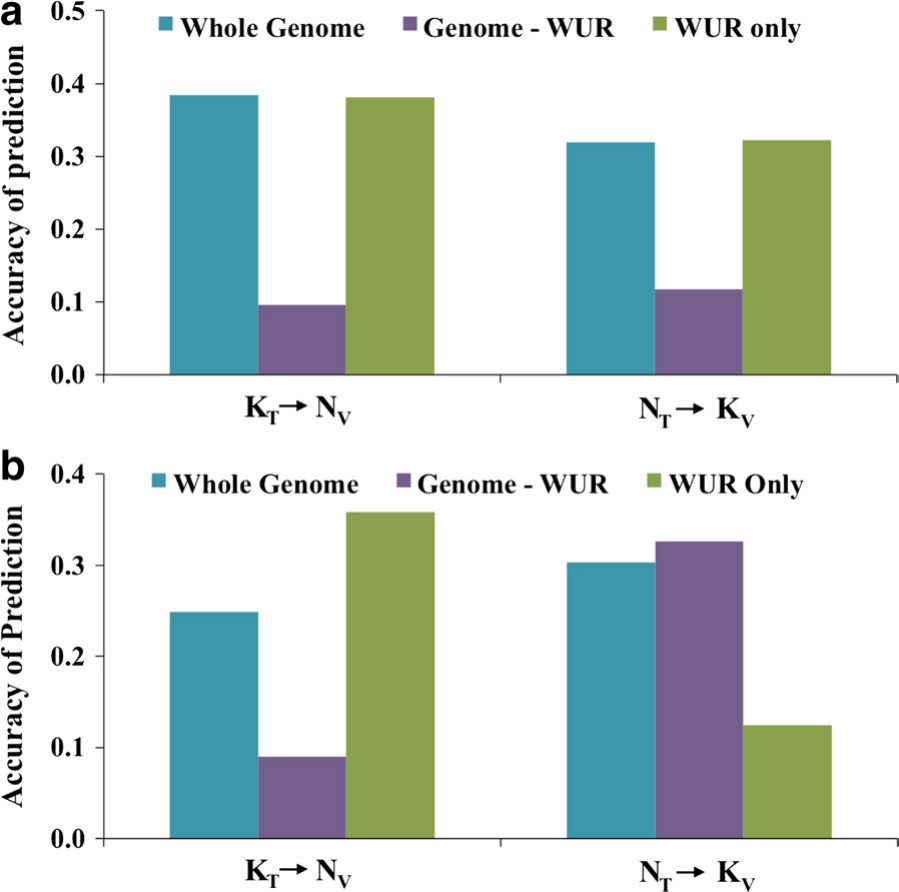
Accuracy of genomic prediction across PRRSV isolates. Accuracy of genomic prediction for VL for different training-validation scenarios, presented as the average correlation between genomic estimated genetic values and adjusted phenotypes divided by the square root of heritability in the validation population for that scenario. Accuracy of genomic prediction across PRRSV isolates for the whole genome, genome minus the WUR genotype (Genome—WUR), and using only the WUR SNP (WUR only) for viral load (**a**) and weight gain (**b**)

Although genomic regions associated with VL were not consistent across PRRSV isolates [12], except for the SSC4 QTL, we found that genomic prediction across PRRSV isolates (black arrows in Fig. 2a) was moderately accurate. When training on the NVSL data and validating on the KS06 data (N_T_ → K_V_), the accuracy of whole-genome prediction was 0.32, while training on the KS06 data predicted GEGV in the NVSL data (K_T_ → N_V_) with an accuracy of 0.38 (Fig. 3a). When we removed the effect of the WUR SNP from the prediction (Genome-WUR), these accuracies reduced to 0.12 and 0.10, respectively (Fig. 3a). Accuracy of prediction using only the WUR SNP (WUR only) was the same as the accuracy obtained when using the whole genome (Fig. 3a). For whole-genome and WUR only predictions, K_T_ → N_V_ was more accurate than N_T_ → K_V_, but K_T_ → N_V_ and N_T_ → K_V_ had equivalent accuracies when using Genome-WUR.

**Fig. 4:**
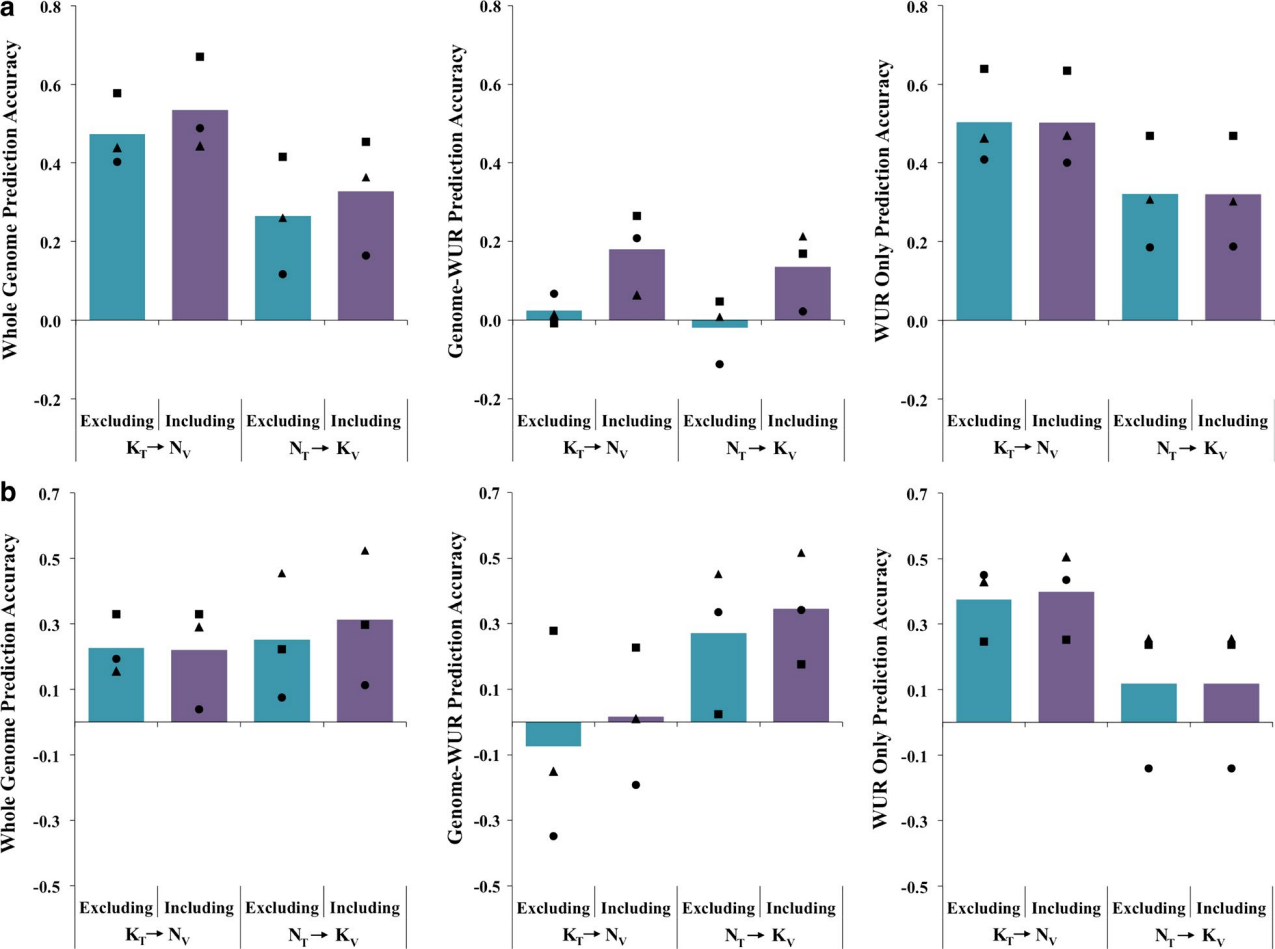
Accuracy of genomic prediction across PRRSV isolates. Accuracy of genomic prediction for different training-validation scenarios, presented as the average correlation between genomic estimated genetic values and adjusted phenotypes divided by the square root of heritability in the validation population for that scenario. Average accuracy of genomic prediction across PRRSV isolates without (Excluding) or with (Including) the validation genetic line in training for viral load (**a**) and weight gain (**b**). Individual points represent the accuracy of prediction for one trial, with genetic lines that are represented in both PRRSV isolates having the same black shape and gray diamonds representing the trials, which were not replicated

**Fig. 5:**
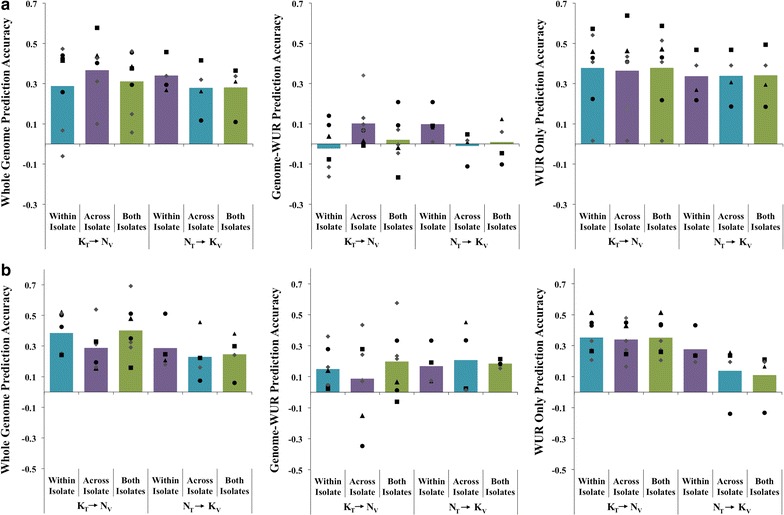
Accuracy of genomic prediction across genetic lines. Accuracy of genomic prediction for different training-validation scenarios, presented as the average correlation between genomic estimated genetic values and adjusted phenotypes divided by the square root of heritability in the validation population for that scenario. Average accuracy of prediction across genetic lines when training and validation are on the same PRRSV isolate, across isolates, or using both isolates for viral load (**a**) and weight gain (**b**). Individual points represent the accuracy of prediction for one trial, with genetic lines that are represented in both PRRSV isolates having the same black shape and gray diamonds representing the trials, which were not replicated

*Effect of genetic relationships between training and validation* Including the validation genetic line in the training population increased accuracy of prediction most for the Genome-WUR prediction (Fig. 4a; genomic prediction accuracies for individual trials are in Fig. 5a). The average prediction accuracy for Genome-WUR when the validation genetic line was excluded from training was 0.005, which increased to an average of 0.16 when the validation genetic line was included in training (Fig. 4a). This suggests that the Genome-WUR predictions are primarily based on genetic relationships. The accuracy of Genome-WUR prediction for line 1 in the NVSL data (indicated by triangles in Fig. 4a) was very low, even with inclusion of related animals in the training population. If the accuracy for this line is ignored, the average accuracy of K_T_ → N_V_ genome-WUR prediction increased from 0.03 to 0.24 when related animals were included in training. Inclusion of related animals in the training population did not have an effect on the accuracy of prediction using only the WUR SNP (Fig. 4a).

#### Prediction across genetic lines with both PRRSV isolates in training

Since the PRRSV mutates rapidly [3], it would be valuable to be able to use data from response to one PRRSV isolate to predict the ability of pigs to respond to another PRRSV isolate. Furthermore, collection of data on response to PRRSV infection is expensive and difficult. Thus, it would be beneficial to use all available data for genomic prediction, resulting in a training dataset consisting of pigs infected with one of several different PRRSV isolates. To assess the effect of PRRSV isolates in the training dataset, we compared genomic prediction across genetic lines within one PRRSV isolate, across PRRSV isolates, and using data from infections with both PRRSV isolates in training, represented by the small blue, large blue, and red arrow, respectively, in Fig. 2b. Results show that whole-genome prediction was moderately accurate for all these scenarios (Fig. 5a). Training on VL in the KS06 data gave the highest accuracy for K_T_ → N_V_ and K_T_ → K_V_ Genome-WUR prediction. On average, whole-genome and WUR-only prediction were not sensitive to the PRRSV isolate used in training. Trial 8 had zero accuracy for WUR-only prediction in the within isolate, across isolate, and both isolates in training scenarios (indicated by a gray diamond). This trial was previously shown to have very low predictive accuracy using only the SSC4 QTL [8]; genotype at the WUR SNP did not have a statistically significant association with VL in that trial [9].

### Genomic prediction of WG

#### Prediction across PRRSV isolates

Accuracies for genomic prediction of WG across PRRSV isolates are in Fig. 3b. Whole-genome or Genome-WUR predictions were most accurate for N_T_ → K_V_. However, for K_T_ → N_V_, WUR only prediction was more than twice as accurate as whole-genome and Genome-WUR prediction (Fig. 3b). The SSC4 QTL was shown to have a large effect on WG in the NVSL data [8] but was not significantly associated with WG in the KS06 data [13]. Bayes-B results showed that the 1-Mb window around the WUR SNP explained 10.4% of genetic variance for WG in the NVSL data, but only 0.05% in the KS06 data (data not shown). GWAS of WG in the KS06 data detected no QTL with large effects but many small effects spread across the genome [12]. Furthermore, genes near SNPs that were moderately associated with WG in the KS06 data were not enriched for metabolic GO terms, unlike those for WG in the NVSL data. The NVSL isolate is more virulent than the KS06 isolate; piglets infected with the KS06 isolate had lower viral load and higher WG than pigs infected with the NVSL isolate [12]. Additional KS06 infection trials may be needed to accurately estimate SNP effects for WG.

*Effect of genetic relationships between training and validation* Including data from the validation genetic line in training had no effect on the average accuracy of genomic prediction of WG for either PRRSV isolate (Fig. 4b), but the average accuracies had large standard errors. Figure 4b shows the trial specific accuracies that were averaged to get each of these estimates. When training on WG from the KS06 trials and predicting trials 1 to 3 or trial 5 of the NVSL data, Genome-WUR prediction yielded negative accuracies but the accuracies of WUR only and whole-genome prediction were positive. Genome-WUR prediction of WG from trial 14 of the KS06 data was moderately accurate, 0.33, but WUR-only prediction accuracy was negative, − 0.14, and whole-genome prediction accuracy was low, 0.07 (Fig. 4b). When training on WG in the NVSL data, whole-genome and Genome-WUR prediction most accurately predicted WG from trial 11 of the KS06 data (Fig. 4b). Accuracies of across-breed prediction of WG for K_T_ → N_V_ using the whole genome ranged from 0.16 to 0.54.

The most notable increase in prediction accuracy due to inclusion of related animals in the training set was for VL in NVSL trial 15 from line 7, for which this increased the Genome-WUR prediction accuracy from − 0.01 to 0.27. Genetic line 7 was separated from all other lines by PCA, which may explain these large increases in accuracy. However, inclusion of related animals in the training set did not substantially increase the prediction accuracy for WG in trial 15. In fact, the largest increases in accuracy due to inclusion of related animals in the training set were for KS06 trial 10, which is from the same line as NVSL trial 15. For this trial 10, inclusion of related animals in the training set led to an increase in accuracy for WG from 0.22 to 0.30 for whole-genome prediction and from 0.02 to 0.18 for Genome-WUR prediction, while WUR-only prediction was not affected by including related animals in the training set.

#### Prediction across genetic lines with both PRRSV isolates in training

Figure 5b shows the average accuracy for WG of whole-genome, Genome-WUR, and WUR-only prediction within isolate, across isolates, and when training on both isolates. Whole-genome and Genome-WUR prediction of WG in the NVSL data was most accurate when NVSL data was included in training (within isolate, N_T_ → N_V_, and both isolates in training, NK_T_ → N_V_). NK_T_ → N_V_ was numerically more accurate than N_T_ → N_V_ for whole-genome and Genome-WUR, 0.38 to 0.40 and 0.15 to 0.20, respectively, but the average accuracies were not statistically significantly different from one another (*t* test *p* value > 0.05). NK_T_ → N_V_ whole-genome prediction was statistically significantly more accurate than K_T_ → N_V_ prediction (0.29 to 0.40; *t* test *p* value = 0.05). No patterns could be discerned from the prediction accuracies of WG in the KS06 data when averaged across all trials. The most notable pattern was for WG in trial 14, in which including both isolates in training was significantly less accurate than within isolate prediction for whole-genome (0.06 to 0.51), Genome-WUR (0.18 to 0.33), and WUR-only prediction (− 0.13 to 0.43). Prediction of WG in KS06 trial 11 was most accurate when WG in the NVSL data was used in training for whole-genome, Genome-WUR, and WUR-only prediction.

## Discussion

The results presented in this study show that even without identifying overlapping genomic regions in GWAS, moderately accurate genomic predictions for host response to PRRS can be obtained across two isolates of the PRRSV. Genomic prediction using the whole genome was more accurate than Genome-WUR or WUR-only prediction for all scenarios for both VL and WG, except when excluding related animals from training in K_T_ → N_V_ for WG, for which using WUR-only was most accurate. For VL, the most accurate genomic prediction scenario involved using the whole genome to predict across PRRSV isolates when the validation genetic line was included in the training data. For WG, the most accurate genomic prediction scenario involved whole-genome prediction with both PRRSV isolates included in the training data.

Overall, genomic prediction of WG was less accurate than genomic prediction of VL. This may, in part, be a result of the higher heritability of VL compared to WG [16–18] and of differences in the effect of the SSC4 QTL on WG for these two PRRSV isolates. Furthermore, growth rate is a common trait that is selected for in the commercial pig industry [19], whereas there is no selection on VL after PRRSV infection. Although the WG used in this study was in piglets infected with PRRSV, as opposed to healthy piglets, prediction of this trait may be affected by prior selection in the industry.

### Genomic prediction method

We used Bayes-B with π = 0.99 for all analyses in this study. Generally, Bayes-B is more appropriate when analyzing a trait with one or several major QTL, because Bayes-B allows for each SNP to have its own variance [1]. In contrast, with Bayes-C and Bayes-Cπ, the fitted SNPs have a common variance [20]. Because the only major QTL was included as a fixed effect in all analyses, it may have been more appropriate to use Bayes-C for prediction with SNPs from the rest of the genome. To investigate this, we repeated the across isolate Genome-WUR analyses using Bayes-C with π = 0.99. Resulting prediction accuracies were not affected for VL but declined by 18% for WG (data not shown). We also analyzed the data using Bayes-C with π = 0, which is equivalent to GBLUP, and found that prediction accuracies were similar to those from Bayes-B with π = 0.99 (data not shown). The Bayes-Cπ method also provided less accurate genomic predictions than Bayes-B with π = 0.99 for both VL and WG. Finally, accuracy was not affected when π in Bayes-B was set equal to the estimate from Bayes-Cπ analysis, compared to using π = 0.99. For VL and WG in the KS06 data, Bayes-Cπ estimates of π were 0.99. For VL and WG in the NVSL data, π was estimated to be 0.56 and 0.42, respectively. Each of these comparisons was made using the Genome-WUR SNP set, as these are the only Bayesian analyses conducted throughout the study. However, the limited datasets prevented a thorough analysis and comparison of alternate methods and priors.

Our estimates of genotypic value include both additive and dominance effects, whereas genomic estimated breeding values (GEBV) are calculated based on only additive effects [1]. For whole-genome prediction in our data, the accuracy of GEBV across PRRSV isolates was the same as the accuracy of GEGV for VL but increased by 30% for WG, from 0.34 to 0.4 for N_T_ → K_V_ and from 0.1 to 0.16 for K_T_ → N_V_. This may be because dominance effects were were small for WG and adding dominance effects with the same prior variance as additive effects added noise to the predictions.

### Genomic prediction across genetic lines

#### Prediction within PRRSV isolate

Prediction of VL across lines within PRRSV isolate was more accurate when using the whole genome than WUR-only prediction, and WUR-only was more accurate than Genome-WUR prediction. Genome-WUR had very little to no predictive ability within isolate for VL, indicating that the effects of the SSC4 QTL contributed most of the prediction accuracy in this scenario. Genome-WUR accuracy was moderately negative for NVSL trial 6, which may be because pigs in this trial were purebred Landrace, while pigs in other trials were crossbreds. The accuracy of within-isolate prediction for VL in NVSL trial 8 was negative for whole-genome and Genome-WUR prediction and near zero for WUR-only (0.02), which is not explained by PCA or the average VL in this trial. The near zero accuracy of WUR-only prediction for trial 8 can be explained by the lack of significant effects of the WUR SNP on VL in this trial [8].

Within PRRSV isolate, prediction of WG was more accurate for the NVSL trials than for the KS06 trials. In the NVSL trials, whole-genome prediction accuracy was higher than WUR-only prediction, and WUR-only prediction was more accurate than Genome-WUR prediction. In the KS06 trials, whole-genome and WUR-only prediction accuracies were equivalent and higher than Genome-WUR prediction accuracies. WUR-only prediction was more accurate in the NVSL trials than in the KS06 trials, which is due to the lack of an effect of the WUR SNP on WG in the KS06 isolate [9]. The effect of WUR in KS06 is smaller than in NVSL data, however it is in the same direction. With this, even the small effect of WUR in the KS06 data predicts the NVSL WG in the same direction with smaller magnitude of effect. However, the large effect of WUR in the NVSL data poorly predicts the KS06 WG, since more of the variance is explained by other regions of the genome. Whole genome predicts well in both directions since the whole-genome prediction is based on addition of the effects from the rest of the genome and the WUR SNP, so if either part has predictive ability, it contributes to the predictive ability of the whole genome.

#### Prediction with related animals in training

On average, inclusion of data from the validation genetic line in the training population increased the accuracy of whole-genome and Genome-WUR prediction for both VL and WG, as expected with greater relationships between the training and validation animals [21–24]. Although other studies have found that genomic prediction across breeds has very low or no accuracy [25–29], we showed that there was some predictive ability across genetic lines in our data when using the whole genome. Although the training and validation data used in that scenario were from different genetic lines, the commercial lines of crossbred pigs used here were likely genetically more similar than the purebred Holstein and Jersey populations used by Hayes et al. [26]. Ibánẽz-Escriche et al. [27], de Roos et al. [25], and Toosi et al. [28] examined the accuracy of genomic prediction across breeds by simulation, which requires many assumptions that are often violated in real data. Another likely explanation for differences in the predictive ability across genetic lines in our study from literature data was the large predictive contribution of the SSC4 QTL. Inclusion of related animals in training increased the accuracy most for Genome-WUR prediction, followed by whole-genome prediction, and least for WUR-only prediction. Therefore, accuracy of genomic prediction across genetic backgrounds without related animals in training and validation may be higher when there are one or several QTL with large effects compared to cases when all QTL effects are small and spread across the genome.

#### Genomic prediction across PRRSV isolates

*Prediction across genetic lines* Prediction accuracies for VL across isolates and genetic lines followed the same pattern as within-isolate prediction across lines, with whole-genome prediction having the highest accuracy, followed by WUR-only prediction, and with Genome-WUR having the least accurate prediction. For across-isolate prediction of WG, accuracy patterns switched between the two isolates; for K_T_ → N_V_, WUR-only prediction was more accurate than whole-genome and Genome-WUR prediction, and whole-genome prediction was more accurate than Genome-WUR prediction; for N_T_ → K_V_, whole-genome prediction was slightly more accurate than Genome-WUR prediction, and the accuracy of Genome-WUR prediction was 51% higher than that of WUR-only prediction. For KS06 trials 10, 11, and 12, the accuracy of WUR-only prediction was low but positive (range 0.19–0.26). However, WUR-only prediction had a negative accuracy (− 0.14) for KS06 trial 14, which can be explained by the fact that direction of the effect of the WUR SNP on WG was opposite (*AA* animals had numerically higher WG than *AB* animals) to its effect in other KS06 trials [9].

*Prediction within isolates, across isolates, and with both isolates in training* For prediction of VL for each PRRSV isolate in validation, average accuracies were similar for within isolate, across isolate, and with both isolates in training for whole-genome and WUR-only prediction. Genome-WUR prediction was most accurate when the KS06 data were used for training. For WG, on average, whole-genome and Genome-WUR predictions were most accurate when NVSL trials were included in training; N_T_ → N_V_ was more accurate than K_T_ → N_V_, N_T_ → K_V_ was more accurate than K_T_ → K_V_, and NK_T_ → K_V_ was more accurate than both N_T_ → K_V_ and K_T_ → K_V_. This may be due to the absence of large effects observed in the GWAS for WG in the KS06 data [12]. For WUR-only prediction of WG in the NVSL data, accuracies were similar for within isolate, across isolate, and with both isolates in training for each validation isolate, with WUR-only prediction of WG having higher accuracy for the NVSL data than for the KS06 data. For WUR-only prediction of WG in the KS06 data, within-isolate prediction was more accurate than across isolate and with both isolates in training. Genotype at the WUR SNP was shown to be associated with WG in the NVSL data but not in the KS06 data, which explains the greater accuracy for WUR-only prediction in the NVSL trials. Effects of the WUR SNP were in the same direction as in the NVSL trials in 3 of 4 KS06 trials. Thus WUR-only prediction had similar accuracy for N_T_ → N_V_ and K_T_ → N_V_.

#### Genomic prediction using SNP subsets based on functional analyses

Although GWAS in this data showed that the most strongly associated regions were inconsistent between these two PRRSV isolates, we found that genes near these SNPs were enriched for several of the same gene ontology (GO) terms. We performed genomic prediction using SNP subsets based on either SNPs that were associated with the trait in the training population or SNPs that were near genes with the enriched GO terms. However, SNP subsets based on association or GO term enrichment information gave lower accuracies than the whole genome (results not shown). Also, excluding the SNPs that were included in the association or GO term enriched categories from whole-genome predictions (results not shown), did not decrease prediction accuracies, which further indicates that these SNP subsets did not contribute significantly to the prediction accuracy. However, in contrast to prediction using only the GO term SNPs, a randomly selected subset of SNPs of the same size had zero predictive accuracy. Jointly, these results indicate that the GO term SNPs are in regions that harbor QTL, but that their effects can also be captured by high-density SNPs outside these regions.

Other statistical methods may be needed to capitalize on the addition of functional annotation of GWAS associations to increase the accuracy of genomic prediction. For example, SNPs could be assigned to subsets based on annotation of genes and different prior distributions of effects could be allowed for each subset, as implemented in the method Bayes-R [30]. In this approach, the whole genome is used for prediction, but the probability of association for SNP subsets is determined by their possible biological relevance. Bayes-N [31] is another Bayesian GWAS and prediction method that could be used in this context. Bayes-N uses a nested model, in which the association of groups of SNPs with the phenotype is analyzed first, followed by associations of SNPs within the associated groups [31].

#### Comparison to previous studies

Our results for within PRRSV isolate Genome-WUR prediction for VL in the NVSL data were similar to the results of Boddicker et al. [8], which used data from trials 1 to 8; however our WUR-only prediction accuracy was lower than obtained by Boddicker et al. [8]. For trial 4, Boddicker et al. [8] showed a negative accuracy when using SNPs across the genome except for the SSC4 QTL (similar to our Genome-WUR scenario), whereas our analysis showed a moderately positive prediction accuracy. There are several possible reasons for these differences. Boddicker et al. [8] used the Bayes-C method of GenSel [32], while we used Bayes-B. Second, we fitted initial age and weight as covariates, while these effects were not fitted in their model. Third, our training dataset included an additional trial of data (trial 15). In addition, Boddicker et al. [8] predicted breeding values, while we estimated genotypic values using both additive and dominance effects for each SNP. Finally, Boddicker et al. [8] adjusted phenotypes in the validation population using estimates of fixed effects obtained from an ASReml analysis of all eight trials, while we used only the validation data to adjust for fixed effects.

## Conclusions

Genomic prediction of response to PRRSV infection was moderately accurate on average, including genomic prediction across PRRSV isolates and across genetic lines. Overall, the Bayes-B method yielded the most accurate genomic predictions. Bayes-C and Bayes-Cπ methods did not increase the accuracy of genomic prediction in this study. Whole-genome prediction across PRRSV isolates and genetic lines was moderately accurate, but accuracy was greatly reduced when SNPs in the SSC4 QTL region were removed from the prediction. The previously identified QTL on SSC4 [6] had a large effect on VL for both PRRSV isolates and on WG for the NVSL isolate [9], and therefore, had a large contribution to prediction accuracy. Greater relationships between training and validation populations had larger effects on genomic prediction accuracy when there were many QTL with small effects for the predicted trait. Especially for VL, inclusion of related animals in the training set yielded the largest increase in the accuracy of Genome-WUR prediction.

Using the whole genome for prediction was most accurate and use of only SNPs shown to be associated with the trait decreased prediction accuracy. Furthermore, SNPs near genes annotated with biologically relevant GO terms had less predictive ability than the whole genome.

This study reveals that the swine industry can use heterogeneous training and validation datasets to implement genomic selection for improved response to PRRSV infection. Genomic selection for either VL or WG is unlikely to lead to piglets that are resistant to PRRSV infection, but will lessen the negative effects of PRRSV infection. Genomic selection for improved response to PRRSV infection will also likely lead to selection of pigs that have improved responses to infection with other viruses or bacteria, increasing the overall robustness of pig.

## Data Availability

The datasets analyzed during the current study are available for research purposes upon request.
